# Kyste épidermoïde du quatrième ventricule: à propos d’un cas

**DOI:** 10.11604/pamj.2017.26.239.8656

**Published:** 2017-04-26

**Authors:** Abderrazzak El Saqui, Mohamed Aggouri, Mohamed Benzagmout, Khalid Chakour, Mohamed El Faiz Chaoui

**Affiliations:** 1 Service Neurochirurgie, CHU Hassan II, Fès, Maroc

**Keywords:** Chirurgie, IRM de diffusion, kyste épidermoïde, quatrième ventricule, Surgery, diffusion MRI, epidermoid cyst, fourth ventricle

## Abstract

Les kystes épidermoïdes sont des tumeurs bénignes rares développées à partir d'inclusions ectodermiques. Ils siègent habituellement au niveau de l'angle ponto-cérébelleux, la région para-sellaire et la fosse temporale. Leur siège au niveau du quatrième ventricule est exceptionnel. Nous rapportons le cas d'une patiente de 47 ans admise pour un syndrome d'hypertension intracrânienne associé à des troubles de la marche. Le diagnostic de kyste épidermoïde du V4 fut évoqué sur les données de l'IRM en séquences de diffusion puis confirmé en per opératoire et en histologie. L'exérèse chirurgicale a été subtotale en raison d'une adhérence de la capsule à la partie supérieure du plancher du V4. Après un recul de 36 mois, la patiente ne manifeste aucun signe de ré-évolution tumorale.

## Abstract (en)

Epidermoid cysts are rare benign tumors developed by ectodermic inclusions. They are usually located at the level of the pontocerebellar angle, the parasellar region and the temporal fossa. They exceptionally develop at the level of the fourth ventricle We report the case of a 47-year old woman admitted for intracranial hypertension syndrome associated with walking disorders. The diagnosis of fourth ventricle epidermoid cyst was evoked based on diffusion MRI data then confirmed intraoperatively and by histologic examination. Subtotal surgical excision was performed due to capsule adhesion to the upper part of the V4 floor. After a 36-month follow-up, the patient showed no signs of tumor recurrence.

## Introduction

Les kystes épidermoïdes, encore appelés cholestéatomes primitifs ou tumeur perlée de Cruveil hier, représentent des tumeurs bénignes rares (environ 2% des tumeurs intracrâniennes primitives), développées à partir d'inclusions ectodermiques. L'angle ponto-cérébelleux représente le siège de prédilection de ces tumeurs. Leur localisation au niveau du quatrième ventricule (V4) est très rare [1]. Seulement 83 cas ont été publiés à ce jour dans la littérature [2]. Nous rapportons le cas d'un volumineux kyste épidermoïde du V4 et nous discutons les particularités clinico-radiologiques, thérapeutiques et évolutives de cette localisation inhabituelle.

## Patient et observation

**Figure 1:**
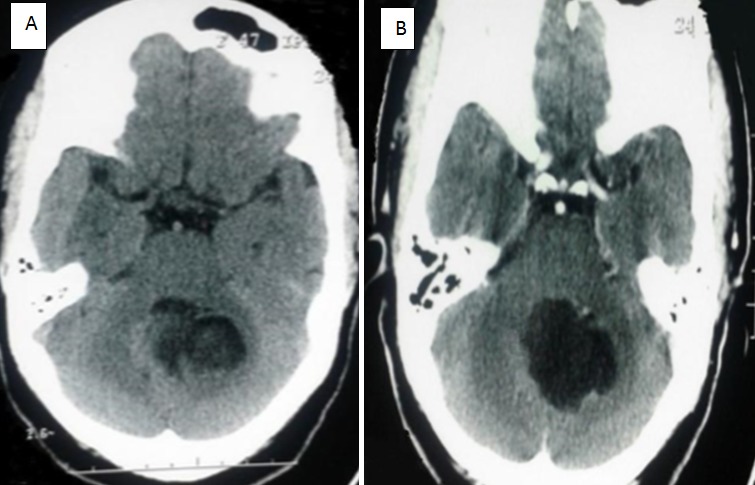
A) TDM cérébrale en coupe axiale sans contrast montrant une lésion hypodense, médiane, hétérogène au niveau de la fosse cérébrale postérieure; B) TDM cérébrale en coupe axiale avec contraste montrant une lésion hypodense de contours irréguliers ne prenant pas le contraste après injection

Il s'agit d'une patiente âgée de 47 ans, sans antécédent pathologique particulier, qui présente depuis un an des céphalées associées à une baisse de l'acuité visuelle (AV). Deux mois avant son admission le tableau clinique s'est aggravé d'une instabilité à la marche. A l'admission, l'examen a trouvé une patiente consciente. La marche était de type ataxique, avec élargissement du polygone de sustentation, sans déficit sensitivomoteur associé. Les réflexes ostéotendineux (ROT) rotuliens étaient pendulaires, et les réflexes cutanéoplantaires (RCP) étaient en flexion. L'acuité visuelle était à 6/10 des deux cotés. Le fond d'œil (FO) a montré un œdème papillaire bilatéral stade II. Devant ce syndrome cérébelleux associé à une hypertension intracrânienne (HTIC), une TDM cérébrale a été demandée montrant une lésion se développant au niveau de la fosse cérébrale postérieure (FCP), médiane, hypodense, aux contours irréguliers, et ne prenant pas le contraste (Figure 1). L'IRM note la présence d'une lésion sous-tentorielle se développant dans le quatrième ventricule qu'elle élargit. La lésion apparaît en hyposignal T1, et en hypersignal T2, ne s'effaçant pas complètement en Flair, sans rehaussement après injection de produit de contraste paramagnétique. Elle était cependant hétérogène en Flair, et en hypersignal en diffusion. La lésion était de contours festonnés, mesurant 40 mm sur 50 mm.

**Figure 2:**
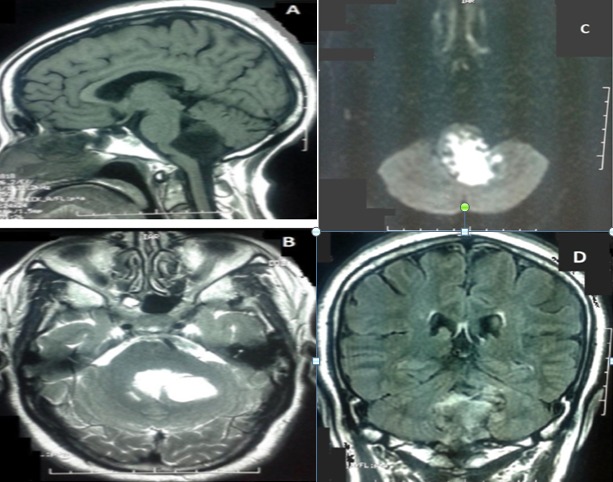
IRM cérébrale en coupe sagittale T1, A) coupe axiale T2; B) diffusion; C) et coronale FLAIR; D) montrant une lésion remplissant la lumière du V4, hypointense T1, hyperintense T2 et en diffusion, de contours irréguliers, évoquant en premier un kyste épidermoïde du V4

**Figure 3:**
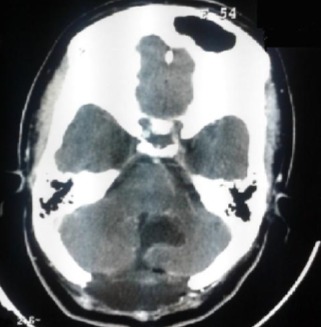
TDM cérébrale C+ post opératoire montrant une craniectomie occipitale médiane avec une méningocèle compliquant l’ablation chirurgicale d’un KE du 4^ème^ ventricule

**Figure 4:**
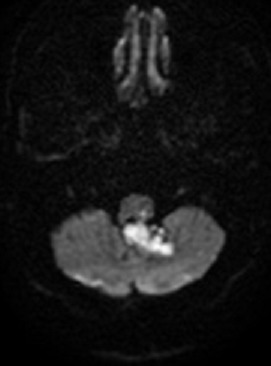
IRM cérébrale coupe axiale en diffusion montrant un résidu tumoral post opératoire au niveau du V4

En avant, elle présentait une empreinte sur le tronc cérébral sans anomalie de signal en son sein, avec effacement des citernes des angles pontocérébelleux, prépontique, et de la grande citerne, et abaissement des amygdales cérébelleuses. Egalement, on a noté une discrète hydrocéphalie sus-jacente (Figure 2). La décision d'un abord direct de la lésion a été prise et la patiente a été opérée par voie postérieure médiane, avec exérèse subtotale de la lésion, après dissection soigneuse de la capsule. L'examen anatomopathologique a montré une formation kystique bordée par un épithélium malpighien régulier reposant sur une fine paroi fibreuse. La lumière comportait des lamelles de kératine. Cet aspect était compatible avec le diagnostic du kyste épidermoïde.Les suites opératoires ont été marquées par l'apparition à J+6 en post opératoire d'un écoulement du liquide céphalorachidien à travers la plaie opératoire avec une tuméfaction rénitente sans signe inflammatoire en regard et sans notion de céphalées associées évoluant dans un contexte de fébricule chiffrée à 38^°^C. Une TDM cérébrale de contrôle réalisée était en faveur d'une méningocèle (Figure 3). La patiente a bénéficié d'une ponction lombaire (PL) qui a objectivé une hyperproteinorachie à 1,3g/l avec une cytologie à 260 leucocytes/mm^3^ à prédominance des polynucléaires neutrophiles (75%). Le diagnostic de méningite purulente postopératoire a été retenu et la patiente a été alors mise sous céftriaxone à dose méningée (100mg/kg/j) avec surveillance des signes cliniques et biologiques de la méningite. La patiente a aussi bénéficié de trois PL déplétives, avec tarissement définitif de l'écoulement du LCR à travers la plaie opératoire. L'évolution a été marquée par une bonne amélioration clinique et biologique. La PL de guérison réalisée à J10 a confirmé la guérison de la méningite. Le suivi de la patiente a noté une nette amélioration de son Syndrome cérébelleux avec une régression des céphalées. L'IRM de contrôle faite 1 an après a montré un résidu tumoral postopératoire (Figure 4). Après un recule de 36 mois, il n'y a aucun signe clinique de reprise évolutive de la tumeur.

## Discussion

Anciennement appelé cholestéatome ou tumeur perlée de Cruveil hier, le kyste épidermoïde est une tumeur rare représentant moins de 2% des tumeurs intracrâniennes [3]. La fréquente latéralité des kystes épidermoïdes serait liée au développement concomitant des vésicules otiques et optiques [4]. La localisation habituelle se rencontre au niveau de l'angle ponto-cérébelleux (40 à 50% des cas selon les séries) [5] ainsi qu'au niveau des régions para-sellaires et temporales. Sa localisation au niveau du quatrième ventricule est très rare [6]. Il s'agit d'une tumeur bénigne secondaire soit à une inclusion ectopique d'éléments ectodermiques au moment de la fermeture du tube neural entre la 3^ème^ et la 5^ème^ semaine de gestation [7], soit, et moins souvent, à une pénétration post-traumatique [2] ou iatrogène [8] de l'épiderme au niveau des espaces sous arachnoïdiens. Malgré sa genèse au cours de la vie intra-utérine la découverte du kyste épidermoïde est tardive entre la 3^ème^ et la 5^ème^ décennie, comme c'est le cas de notre patiente. Sur le plan clinique, le syndrome cérébelleux est la manifestation la plus fréquente, alors que le syndrome d'hypertension intracrânienne est moins fréquent, étant donné que l'hydrocéphalie sus tentorielle est d'apparition tardive et ne se voit que dans moins de 50% des cas [9]. De même la croissance très lente de la tumeur et la probable persistance d'espace d'écoulement du LCR entre la capsule et les parois du ventricule explique l'absence de corrélation entre l'importance du volume tumoral et la présence d'hydrocéphalie au moment de découverte de la tumeur [10]. L'extension vers la citerne ponto-cérébelleuse par l'intermédiaire des trous de Luschka, se traduit par une atteinte des nerfs crâniens (nerfs mixtes, paquet acoustico-facial, nerf trijumeau). L'aspect IRM des kystes épidermoïdes est identique quelle que soit leur localisation. Ils sont isointenses en T1 et hyperintenses en T2, avec des limites nettes mais irrégulières, sans 'dème périlésionnel ni de prise de contraste. En effet, le signal est souvent inhomogène; il peut être variable en intensité en fonction du contenu protidique de la tumeur.

Des formes atypiques ont été rapportées, avec une masse spontanément hyperintense en T1 et hypointense en T2, probablement du fait de la présence de calcifications et d'un contenu protidique élevé. Les problèmes de diagnostic différentiel avec les kystes arachnoïdiens et les kystes tumoraux sont contournés grâce à l'aspect hétérogène en séquence Flair, l'augmentation du signal en séquence de diffusion et surtout à l'aspect hyperintense et hétérogène en séquence CISS-3D [11]. L'analyse histologique des kystes épidermoïdes est la même, quelle que soit la localisation intracérébrale. Sur le plan thérapeutique, l'exérèse totale du kyste et de sa capsule reste le seul garant d'une guérison définitive. Cependant, et comme dans notre cas, l'intime adhérence de la capsule au plancher du V4 limite cette option vu les risques neurologique et vital encourus. Ainsi, et sur une revue de la littérature réalisée par Tancredi A. et collaborateurs [9] concernant 66 patients opérés pour un kyste épidermoïde du V4 entre 1974 et 2003, l'exérèse totale n'a été pratiquée que dans 30% des cas. L'évolution postopératoire est habituellement simple; toutefois, une méningite chimique peut survenir et engendrer une hydrocéphalie communicante, dont la prévention passe par l'exérèse totale tant que possible, l'éviction de la dispersion du contenu du kyste en per-opératoire, ainsi que l'irrigation du foyer opératoire par de l'hydrocortisone voire l'administration en postopératoire de la dexamethasone [12]. Dans le cadre de surveillance postopératoire, l'imagerie de diffusion permet d'établir le caractère complet ou non de l'exérèse. En cas de résidu tumoral, une surveillance annuelle par IRM permet d'évaluer le potentiel évolutif du résidu [8].

## Conclusion

Le kyste épidermoïde du 4^ème^ ventricule est une tumeur bénigne rare dont le pronostic est le plus souvent favorable. L'IRM de diffusion reste l'examen clé en matière de diagnostic positif et de surveillance postopératoire. L'exérèse chirurgicale totale est conditionnée par la présence d'une portion capsulaire plus ou moins adhérente au plancher du V4. La chirurgie reste la seule mesure thérapeutique disponible.
